# Continuous assessment of cardiac output during exercise using real time flow with fast GPU reconstruction

**DOI:** 10.1186/1532-429X-14-S1-P232

**Published:** 2012-02-01

**Authors:** Grzegorz T Kowalik, Jennifer A Steeden, Bejal Pandya, David Atkinson, Andrew M Taylor, Vivek Muthurangu

**Affiliations:** 1 Centre for Cardiovascular MR, UCL, Institute of Cardiovascular Science, London, UK; 2 UCL Department of Medical Physics & Bioengineering, Centre for Medical Image Computing, London, UK

## Background

The haemodynamic response to exercise is an important health marker. Phase contrast MR (PCMR) is an accurate method of assessing cardiac output and novel real-time sequences allow assessment of flow during exercise. However, such sequences often have time-consuming reconstruction algorithms, which prevent continuous cardiac output monitoring during exercise.

Graphical processing units (GPU) with multiple processors offer the possibility of performing extremely fast reconstruction of real-time MR data. Such reconstructions would make continuous assessment of cardiac output during exercise possible and this projects aim to validate this approach.

## Methods

Twenty healthy volunteers underwent aortic flow assessment using spiral SENSE real-time PCMR (12 interleaves, 4-fold SENSE acquisition, 43ms temporal resolution, 13980 frames, acquisition time 10 minutes). Aortic flow was measured continuously during a ramped cycle exercise (2-16 W, increased every minute by 2 W). The online iterative SENSE reconstruction was implemented in CUDA and performed on a bi-directionally networked computer (GPU card: NVidia Geoforce 480).

The GPU reconstruction task was split into batches of sixty frames with incoming data being buffered on CPU RAM, which allowed overlapping between data transmission and reconstruction.

Aortic flow images were analysed using a semi-automated optic-flow registration and segmentation algorithm. The algorithm was implemented as a multithreaded application to speed-up processing time.

## Results

The new GPU implementation was tested and compared against our original CPU version. A single batch of 60 flow measurements was retrospectively reconstructed with both versions. GPU implementation showed negligible bias in stroke volume of -0.4ml and good limits of agreement -1.9 to 1.2ml.

A single batch of 60 frames reconstructed 7.7 times faster on the GPU than CPU (15.2 if time required for data transmission is excluded). Total reconstruction time for the 13980 frames (including transmission and buffering) was ~626s. Thus, data was available for use ~9s after the scan finished. This is a 556 times speed-up compared to the estimated CPU reconstruction (of ~83min).

**Figure 1:**
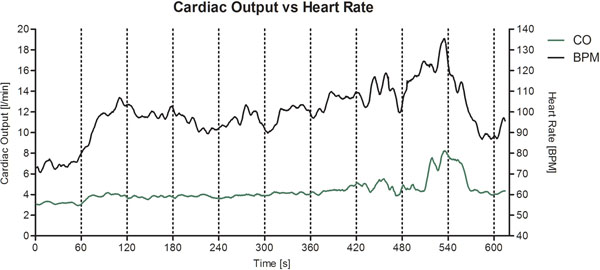


Fig 1 shows a representative plot of continuous heart rate and cardiac output changes during exercise in one volunteer.

## Conclusions

Continuous assessment of flow during exercise could provide a novel way of assessing hemodynamic responses in patients. Unfortunately, real-time MR sequences have long reconstructions that would leave the clinician waiting for more than an hour for the data. We have shown that by performing a GPU reconstruction, the data can be used within seconds of the acquisition finishing. Furthermore using this technique we have been able to show the expected response to continuous exercise in healthy volunteers.

## Funding

British Heart Foundation.

